# Assessing the physiological effect of non-driving-related task performance in conditionally automated driving systems: A systematic review and meta-analysis protocol

**DOI:** 10.1177/20552076231174782

**Published:** 2023-05-08

**Authors:** Rory Coyne, Leona Ryan, Mohamed Moustafa, Alan F Smeaton, Peter Corcoran, Jane C Walsh

**Affiliations:** 1 School of Psychology, 8799 University of Galway, Galway, Ireland; 2 School of Engineering, 8799 University of Galway, Galway, Ireland; 3 School of Computing, 8818 Dublin City University, Dublin, Ireland; 4 Department of Electrical and Electronic Engineering, 8799 University of Galway, Galway, Ireland

**Keywords:** Automated driving, physiology, psychophysiology, driver behaviour, advanced driver assistance systems, task performance

## Abstract

**Background:**

Level 3 automated driving systems involve the continuous performance of the driving task by artificial intelligence within set environmental conditions, such as a straight highway. The driver's role in Level 3 is to resume responsibility of the driving task in response to any departure from these conditions. As automation increases, a driver's attention may divert towards non-driving-related tasks (NDRTs), making transitions of control between the system and user more challenging. Safety features such as physiological monitoring thus become important with increasing vehicle automation. However, to date there has been no attempt to synthesise the evidence for the effect of NDRT engagement on drivers’ physiological responses in Level 3 automation.

**Methods:**

A comprehensive search of the electronic databases MEDLINE, EMBASE, Web of Science, PsycINFO, and IEEE Explore will be conducted. Empirical studies assessing the effect of NDRT engagement on at least one physiological parameter during Level 3 automation, in comparison with a control group or baseline condition will be included. Screening will take place in two stages, and the process will be outlined within a PRISMA flow diagram. Relevant physiological data will be extracted from studies and analysed using a series of meta-analyses by outcome. A risk of bias assessment will also be completed on the sample.

**Conclusion:**

This review will be the first to appraise the evidence for the physiological effect of NDRT engagement during Level 3 automation, and will have implications for future empirical research and the development of driver state monitoring systems.

## Introduction

Automated driving systems (ADS) represent an innovative means of leveraging artificial intelligence for the assistance or performance of driving-related tasks. In the context of ADS, the extent of a system's automation can be categorised across a number of levels, ranging from Level 0 (no automation) to Level 5 (full automation).^1^ Level 3, or conditional automation, is the highest level currently available at the consumer level and can be defined as the continuous performance of all driving-related tasks within a specified operational design domain (ODD). In Level 3 automation, when the system encounters a scenario outside of its ODD (e.g. missing lane markings), a takeover request is issued to the user, who must then resume control of the vehicle. As such, while the user can delegate all driving-related tasks to the system in Level 3, they must remain ready to resume control.

While the handover of responsibility of driving-related tasks to artificial intelligence affords greater opportunities to promote driver safety and wellbeing, in Level 3 automation, interactions related to transitions of control can be problematic. This is because as the level of vehicle automation increases, the driver is increasingly removed from the system control loop, making the transition back to manual driving, when needed, very difficult.^2,3^ During prolonged periods of automated driving, drivers may experience what is referred to as ‘passive fatigue’ by May and Baldwin^4^ due to the monotonous nature of simply monitoring the road without driving. As fatigue increases, drivers may even experience a state of drowsiness.^5^ They may also become distracted from monitoring the road as their attention shifts towards non-driving-related tasks (NDRTs).

An NRDT can be defined as any auxiliary task which does not directly or indirectly assist in the performance of the driving task. Drawing on findings from real-world observational research, users of conditionally ADS display a greater propensity to engage in NDRTs.^6^ Hecht et al.^7^ found that drivers free to perform NDRTs during a 1-hour period of Level 3 automated driving most commonly engaged in mobile phone use (75% of their sample), reading (60%) or browsing a tablet (50%). NDRTs are commonly used to induce psychological and cognitive states in the driving environment (e.g. stress, cognitive load) in a laboratory setting. The freedom to engage in NDRTs is seen as a major opportunity afforded by automated driving.^8^

A number of recent reviews have found evidence that engaging in an NDRT results in worse takeover performance, particularly when there are overlapping resource demands between the NDRT and driving task – that is, when the NDRT has a visual or a manual component.^9–12^ According to the out-of-the-loop performance problem,^13^ as the level of vehicle automation increases, drivers display a diminished capability to respond to sudden or urgent takeover requests from the system. Being ‘out of the loop’ is characterised by a situation where the user is not actively involved in the control of a system and is not monitoring the environment. This leads to decrements in the user's situation awareness. A classic definition of situation awareness is “knowing what's going on so you can figure out what to do”.^14^ Being out of the loop also results in a delay in the user's reaction time to critical events, particularly as they take up NDRTs during automated driving.^15^ De Winter et al.^16^ found in their review that NDRT engagement during automated driving can result in poorer situation awareness than during manual driving. This understandably could have serious implications for user safety during conditionally automated driving, when the driver is assumed to be ready to take over as needed.

Malleable attentional resources theory^17^ suggests NDRTs may actually mitigate increasing fatigue due to automation. This theory proposes that attentional resources shrink to meet situational demands, and so performing an NDRT could combat such a shrinkage and prevent the onset of fatigue. In their review, De Winter et al.^18^ found that a monotonous automated drive led to slower reaction times compared with manual driving when drivers are experiencing a state of drowsiness. Miller et al.^19^ also suggested that NDRTs could be used to reduce drowsiness. It is therefore possible that NDRT performance during automated driving could lead to conservation of attentional resources (and consequently, one's ability to respond) in certain scenarios.

In addition to simply engaging in an NDRT during automated driving, the modality of the NDRT seems to have an effect on driver performance. Wandtner et al.^20^ conducted a driving simulator study with several critical takeover scenarios and found that when drivers were performing an NDRT that was both visual and manual, performance degraded the most. As driving is also both a visual and manual task, the authors concluded that NDRTs with resource demands that overlap with the driving task can result in interference with driving performance. In their review, Zhang et al.^10^ found that longer takeover times were associated with performing a visual NDRT – a similar finding was observed in reviews by Merlhiot et al.^11^ and McDonald et al.^12^ with respect to handheld tasks. Wickens’ multiple resource theory^21^ accounts for how performance interference is greater among two tasks with competing resources, compared with two tasks that differ in some way. In the context of automated driving, the idea that some NDRTs interfere with driving more than others could have implications for safety.

Meinlschmidt et al.^22^ conducted a systematic review to examine the effect of ADS engagement on drivers’ physiological responses. The authors found that increased electrodermal and masseter electromyography activity and decreased heart rate were observed when ADS was engaged. However, these findings were based on only four studies (*N*  =  194 participants). As recent technological advancements have made it possible to study driver performance in immersive, high-fidelity driving simulators with greater ease, there is a pool of recent research which has yet to be synthesised.^22–25^ Furthermore, it will soon become a requirement for all ADS to be equipped with a driver monitoring system that can track the psychological or cognitive state of the driver and make an assessment about the driver's fitness to drive.^26^ This will rely heavily on the collection of physiological data; hence these measures are of particular interest.

It is clear from previous systematic reviews in this area^9–12^ that NDRT engagement can have consequences for takeover performance, and there is some evidence to suggest this effect may vary depending on specific NDRT attributes. However, this is the first systematic review that will directly attempt to synthesise the evidence concerning the effect of NDRTs on drivers’ physiological measures. This work is of particular importance to the future of conditionally automated driving, in which assessments will be made about a driver's readiness to drive through physiological data collection. This review will also expand on other reviews in this area that examined the effect of mental states during automated driving^11,27^ by looking at physiological measures, which can be used as an index of underlying mental states.

### Objectives

The central objective of the present systematic review and meta-analysis is to examine the effect of NDRT engagement on drivers’ physiological responses in Level 3 ADS. The secondary objective of this review, guided by Wickens’ multiple resource theory, is to examine the effect of visual NDRTs versus nonvisual NDRTs, and manual NDRTs versus nonmanual NDRTs, on drivers’ physiological responses.

## Methods

The present systematic review and meta-analysis was submitted for registration to the International Prospective Register of Systematic Reviews (PROSPERO) on July 1^st^, 2022 and was registered on July 12^th^, 2022. The methods chosen for this review were guided by the Preferred Reporting Items for Systematic reviews and Meta-Analyses for Protocols (PRISMA-P).^28^ Any amendments to the protocol for this review will be documented in accordance with the Preferred Reporting Items for Systematic Reviews and Meta-Analyses (PRISMA)^29^ statement.

### Information sources and search strategy

The electronic databases MEDLINE (Ovid), EMBASE (Elsevier), Web of Science (Core Collection), PsycINFO (Ovid), Compendex (Elsevier) will be searched for records for the purpose of this review. These databases were selected in consultation with a librarian information specialist. To minimise publication bias, the electronic databases Compendex and PsycExtra will be used to search for grey literature. The databases will be queried using the search strategy developed for this review (see Appendix A), and the following filters will be applied: English language only, and records existing from January 1^st^, 2012 to the date of search (July 29^th^, 2022). Forward and backward citation searches will be conducted with the sample of eligible studies obtained from the database and grey literature search. Where a full-text article is not accessible, study authors will be contacted to request the full-text. A cut-off period of 2 weeks will be provided, whereby the full-text will be considered inaccessible if there is no author response 2 weeks after it is requested. A follow-up database search will be conducted on November 1^st^, 2022, using the identical search strategy and filters applied during the initial search, to ensure that all relevant and up-to-date records and included in this review. Given that this is a particularly novel and fast-progressing area of research, it was decided that a follow-up search was warranted.

The search strategy for this review was developed by identifying search terms used in Meinlschmidt et al.,^16^ upon which this review is expanding by specifically examining NDRT engagement and synthesising a larger pool of more recent data. A librarian information specialist was also consulted with to develop the search strategy. Three concept blocks were constructed: search terms related to automobile driving, terms related to automation, and terms related to physiological activity. Where possible, search terms were mapped on to relevant subject headings and supplemented with free terms. Subject headings were chosen by conducting a scoping search to identify headings used by studies that would be eligible for inclusion in this review. The search terms within each concept block were combined using the Boolean operator OR, and the three concept blocks were then combined using the AND operator. Appendix A contains a list of all search terms and syntax that will be used in each database for this review.

### Eligibility criteria

The eligibility criteria for this review were developed in accordance with the patient, intervention, comparison, outcome, studies (PICOS) model.^30^

#### Population

Participants from eligible studies will include adults aged 18 years or older, holding a full driving license at the time of the study. Studies that recruit participants that either do not hold a full driving license at the time of participation, or are below 18 years of age, will be excluded from the review.

#### Interventions

Studies which use either an on-road vehicle, fixed-base driving simulator, or fixed-base off-road vehicle with an audio/visual display, capable of performing or simulating Level 3 (conditional automation) will be included. Studies which use a vehicle or simulator that can perform another level of automation (Level 1, 2, 4 or 5), and do not include a vehicle or simulator capable of performing Level 3 automation will be excluded. Studies that use manual driving only will be excluded. Studies must also use at least one type of NDRT in addition to the primary driving task. The categorisation of NDRTs for this review will be based largely on a recent overview by Naujoks et al.,^31^ but broadly speaking, they can be categorised as being either visual or nonvisual in terms of their modality of presentation, and manual or nonmanual in terms of task input. Studies which use Level 3 automation but do not include an NDRT will be excluded. Studies which include only additional tasks that relate to the primary driving task (e.g. pressing a button to toggle vehicle automation on/off) will be excluded.

#### Comparators

Studies which include either a within-subjects baseline condition, or a between-subjects comparison group with no NDRT involvement will be included. This is a necessary criterion for inclusion, as the presence of such a condition provides a reference value against which the effect of the NDRT can be compared. Studies which do not feature either a within-subjects or between-subjects comparator will be excluded. Studies which compare the differential effects of various NDRTs, but do not feature either a within-subjects or between-subjects comparator, will be excluded.

#### Outcomes

Eligible studies will measure at least one peripheral physiological parameter that is directly measurable by psychophysiological monitoring equipment. Appendix B contains a list of potential physiological measures that we expect to see in included studies. Studies which measure biochemical parameters only (e.g. cortisol, interleukin 6, C-reactive protein) will be excluded. Studies which use self-report measures only will be excluded. Eligible studies must measure at least one peripheral physiological parameter both during NDRT engagement and during either a within-subjects baseline or between-subjects comparison condition.

#### Types of study

Empirical studies that use either a vehicle or driving simulator capable of performing Level 3 (conditional) automation and employ at least one type of NDRT will be included. Eligible studies will compare the effect of NDRT involvement on physiological responses with either a within-subjects baseline condition, or a between-subjects comparison group. Grey literature (conference proceedings, unpublished dissertations) will be included. Articles which use qualitative methods only (such as interviews or focus groups) will be excluded. Editorials, theoretical papers, and review papers will be excluded. Studies not published in English will be excluded. Studies published prior to January 1^st^, 2012 will be excluded.

### Article screening

The results of the electronic database search will be merged and deduplicated in EndNote 20 citation management software. Following deduplication, the results will be exported to Rayyan for screening. Article screening will take place in two stages. In stage 1, one independent reviewer (RC) will assess the titles and abstracts of the total deduplicated results, and a second reviewer (LR) will assess a random sample (as recommended by Shoukri^32^) of 20% to check for inter-observer agreement. In stage 2, RC will screen the full-text articles of all records deemed to have met the eligibility criteria at stage 1. LR will again screen a random sample of 20% of the full-text articles for this sample. If there is disagreement between RC and LR at either stage regarding whether a record meets the eligibility criteria, a third reviewer (MM) will be consulted with, and their decision will determine that study's eligibility. The results of the screening process will be presented in a PRISMA flow diagram, along with a rationale for exclusion of articles at each stage. Cohen's kappa statistic will be calculated at stage 1 and stage 2 to assess the inter-observer agreement among authors.

### Data extraction

One reviewer (RC) will extract relevant data from the final sample of eligible studies, using a pre-determined electronic data extraction form that will be managed using Microsoft Word (see Appendix C). The data that will be extracted will comprise: (i) bibliographic information (study title, authors, year of publication, country of origin), (ii) study characteristics (study design, theoretical background/conceptual models mentioned), (iii) participant characteristics (age, gender, level of experience with ADS), (iv) information about the experiment (the type of vehicle or simulator used, vehicle speed, the content, modality and duration of NDRT employed, and environmental factors such as traffic density or weather condition), (v) outcome measures (type of physiological outcomes, method of measurement), (vi) main findings (results with respect to the effect of NDRT engagement on physiological outcome measures) and (vi) limitations acknowledged by the author(s).

### Critical appraisal

For the purpose of critical appraisal of the final sample of included studies in the review, the risk of bias in nonrandomised studies of interventions (ROBINS-I) tool^33^ will be used. The ROBINS-I tool assesses risk of bias across seven domains, which are broadly categorised into pre-intervention domains (bias due to confounding, bias in selection of participants), at intervention (bias in classification of interventions) and post-intervention domains (bias due to deviations from intended interventions, bias due to missing data, bias in outcome measurement, bias in selection of reporting of results). A six-step process is taken for study assessment. The first steps involve specifying the research question, the outcome and result being addressed. The assessor must then examine whether and how confounders were addressed with respect to the results. Subsequently, they must address a series of signalling questions for each domain provided by Sterne et al.^33^ Finally, the assessor will formulate a risk of bias judgement for each domain and make an overall judgement of the risk of bias (low risk, moderate risk, serious risk, critical risk, or no information).

### Data synthesis and analysis

To address the central objective of this review, the findings with respect to the effect of NDRT engagement on drivers’ physiological responses in Level 3 ADS will be synthesised in a series of meta-analyses. The data will be meta-analysed by outcome measure in R using the *meta* package.^34^ The meta-analyses will assess the effect of NDRT engagement on physiological parameters, in comparison to either a within-subjects baseline condition, or a between-subjects control condition.

Risk ratio (RR), mean difference (MD) or standardised mean difference (SMD) between conditions will be calculated for each measure across all included studies in R using extracted data from included studies and expressed at the 95% confidence interval. Where critical statistical information is not available in-text, but results are presented in a graph format, the online package WebPlotDigitizer will be used to extract statistical information from the graph. This online package has previously demonstrated high levels of intercoder reliability and validity.^35^

The level of heterogeneity between-studies will be assessed using the Higgins *I*² statistic. In the presence of moderate heterogeneity (Higgins *I*² > 50%), random-effects model will be used, otherwise a fixed-effects model will be used. In the case of high heterogeneity (Higgins *I*² > 75%) a narrative synthesis of the data will be conducted. The results of a series of meta-analyses will be presented using forest maps stratified by the type of physiological measure assessed in included studies. For the purpose of exploratory analysis, a series of meta-analyses will be conducted to examine the effect of NDRT modality (visual NDRTs vs nonvisual NDRTs, manual NDRTs vs nonmanual NDRTs) on drivers’ physiological responses.

## Results

The results of the database search will be presented in a PRISMA flow diagram, which will include the number of articles excluded at each stage of the screening process, and the reasons for exclusion. The results of the data extraction from the final sample of included studies will first be presented in a table of results, and key study characteristics will be reported in-text. This descriptive information will include information on the population, study design and outcome measures of the final sample of included studies. Following this, the results of the meta-analysis will be presented, followed by the results of the risk of bias assessment. It is anticipated that NDRT performance will lead to greater physiological activation, compared with when no NDRT is performed. Additionally, in line with Wicken's multiple resource theory, we expect that NDRTs with resource demands which overlap with the driving task (i.e. manual and visual tasks) will lead to greater physiological activation, compared with NDRTs with resource demands that do not overlap with the driving task.

## Conclusion

In summary, the present systematic review and meta-analysis will be the first to critically assess the effect of NDRT engagement on drivers’ physiological responses in Level 3 ADS. While previous reviews have shown that engaging in NDRTs can have a negative effect on driver performance metrics, far less is known concerning how secondary tasks impact the physiological state of the driver. This information is central to the development of driver monitoring systems which can continuously track the psychological or cognitive state of the user, and issue pre-emptive warnings when a target state reaches critical levels. The findings of the review are likely to provide several insights for researchers in this area. For one, it will elucidate the extent to which task performance affects driver arousal levels during conditionally automated driving. Such information will be helpful in efforts to measure complex constructs such as stress, fatigue and cognitive load in the in-cabin setting.

## Supplemental Material

sj-docx-1-dhj-10.1177_20552076231174782 - Supplemental material for Assessing the physiological effect of non-driving-related task performance in conditionally automated driving systems: A systematic review and meta-analysis protocol Click here for additional data file. Supplemental material, sj-docx-1-dhj-10.1177_20552076231174782 for Assessing the physiological effect of non-driving-related task performance in conditionally automated driving systems: A systematic review and meta-analysis protocol by Rory Coyne, Leona Ryan, Mohamed Moustafa, Alan F Smeaton, Peter Corcoran and Jane C Walsh in DIGITAL HEALTH
