# Supplementary material for: Assessing the physiological effect of non-driving-related task performance in conditionally automated driving systems: A systematic review and meta-analysis protocol
Source: Digit Health. 2023 May 8;9:20552076231174782. doi: 10.1177/20552076231174782 (PMC10176551; doi:10.1177/20552076231174782)
Supplement: sj-docx-1-dhj-10.1177_20552076231174782 - Supplemental material for Assessing the physiological effect of non-driving-related task performance in conditionally automated driving systems: A systematic review and meta-analysis protocol [file sj-docx-1-dhj-10.1177_20552076231174782.docx]

**Appendix A**

Search strategy for each database including full list of search terms and syntax.

Database: MEDLINE (Ovid)

1 Automobiles/

2 automobil$.ti,ab,kf.

3 Automobile Driving/

4 Accidents, Traffic/

5 Distracted Driving/

6 driver impairment.ab,ti,kf.

7 driver behavio*r.ab,ti,kf.

8 driving performance.ab,ti,kf.

9 human factors.ab,ti,kf.

10 driver distraction.ab,ti,kf.

11 driver take-over.ab,ti,kf.

12 driver takeover.ab,ti,kf.

13 1 or 2 or 3 or 4 or 5 or 6 or 7 or 8 or 9 or 10 or 11 or 12

14 Automation/

15 Man-Machine Systems/

16 Artificial Intelligence/

17 Autonomous Vehicles/

18 autonomous vehicl$.ab,ti,kf.

19 autonomous driving.ab,ti,kf.

20 human automation interaction.ab,ti,kf.

21 non-driving related task$.ab,ti,kf.

22 vehicle automat$.ab,ti,kf.

23 transition of control.ab,ti,kf.

24 conditional$ automat$ driv$.ab,ti,kf.

25 automat$ driv$.ab,ti,kf.

26 takeover request.ab,ti,kf.

27 take over request.ab,ti,kf.

28 driv$ monitor$ system$.ab,ti,kf.

29 high$ automat$ driv$.ab,ti,kf.

30 automat$ driv$ system$.ab,ti,kf.

31 14 or 15 or 16 or 17 or 18 or 19 or 20 or 21 or 22 or 23 or 24 or 25 or 26 or 27 or 28 or 29 or 30

32 Fatigue/

33 fatigue$.ab,ti,kf.

34 "Task Performance and Analysis"/

35 task performance.ab,ti,kf.

36 task engage$.ab,ti,kf.

37 Cognition/

38 Stress, Physiological/ or Stress, Psychological/

39 stress.ab,ti,kf.

40 Eye-Tracking Technology/

41 eye-tracking.ab,ti,kf.

42 Galvanic Skin Response/

43 galvanic skin response.ab,ti,kf.

44 skin conduct$.ab,ti,kf.

45 electrodermal.ab,ti,kf.

46 Heart Rate/

47 Attention/

48 attention.ab,ti,kf.

49 Psychomotor Performance/

50 Reaction Time/

51 Awareness/

52 aware$.ab,ti,kf.

53 Risk-Taking/

54 Boredom/

55 bored$.ab,ti,kf.

56 Fixation, Ocular/

57 fixation.ab,ti,kf.

58 Multitasking Behavior/

59 Workload/

60 workload.ab,ti,kf.

61 Blood Pressure/

62 blood pressure.ab,ti,kf.

63 Respiration/

64 respiration.ab,ti,kf.

65 Electromyography/

66 electromyograph$.ab,ti,kf.

67 Psychophysiology/

68 psychophysiolog$.ab,ti,kf.

69 Physiology/

70 physiolog$.ab,ti,kf.

71 drows$.ab,ti,kf.

72 vigila$.ab,ti,kf.

73 electrocardiograph$.ab,ti,kf.

74 electroencephalograph$.ab,ti,kf.

75 pupil.ab,ti,kf.

76 blink.ab,ti,kf.

77 eyeblink.ab,ti,kf.

78 PERCLOS.ab,ti,kf.

79 32 or 33 or 34 or 35 or 36 or 37 or 38 or 39 or 40 or 41 or 42 or 43 or 44 or 45 or 46 or 47 or 48 or 49 or 50 or 51 or 52 or 53 or 54 or 55 or 56 or 57 or 58 or 59 or 60 or 61 or 62 or 63 or 64 or 65 or 66 or 67 or 68 or 69 or 70 or 71 or 72 or 73 or 74 or 75 or 76 or 77 or 78

80 13 and 31 and 79

81 limit 80 to (english language and yr="2012 -Current")

Database: Compendex (Elsevier)

1. (Automobiles CV)

2 ((automobil* TI) OR (automobil* AB) OR (automobil* KY))

3 (Automobile Drivers CV)

4 (Highway accidents CV)

5 ((driver impairment TI) OR (driver impairment AB) OR (driver impairment KY))

6 ((driver behavio*r TI) OR (driver behavio*r AB) OR (driver behavio*r KY))

7 ((driving performance TI) OR (driving performance AB) OR (driving performance KY))

8 ((human factors TI) OR (human factors AB) OR (human factors KY))

9 ((driver distraction TI) OR (driver distraction AB) OR (driver distraction KY))

10 ((driver take-over TI) OR (driver take-over AB) OR (driver take-over KY))

11 ((driver takeover TI) OR (driver takeover AB) OR (driver takeover KY))

12 1 or 2 or 3 or 4 or 5 or 6 or 7 or 8 or 9 or 10 or 11

13 (Automation CV)

14 (Man Machine Systems CV)

15 (Artificial Intelligence CV)

16 (Autonomous Vehicles CV)

17 ((autonomous vehicl* TI) OR (autonomous vehicl* AB) OR (autonomous vehicl* KY))

18 ((autonomous driving TI) OR (autonomous driving AB) OR (autonomous driving KY))

19 ((human automation interaction TI) OR (human automation interaction AB) OR (human automation interaction KY))

20 ((non driving related task* TI) OR (non driving related task* AB) OR (non driving related task* KY))

21 ((non-driving related task* TI) OR (non-driving related task* AB) OR (non-driving related task* KY))

22 ((vehicle automat* TI) OR (vehicle automat* AB) OR (vehicle automat* KY))

23 ((transition of control TI) OR (transition of control AB) OR (transition of control KY))

24 ((conditional* automat* driv* TI) OR (conditional* automat* driv* AB) OR (conditional* automat* driv* KY))

25 ((automat* driv* TI) OR (automat* driv* AB) OR (automat* driv* KY))

26 ((takeover request* TI) OR (takeover request* AB) OR (takeover request* KY))

27 ((take over request* TI) OR (take over request* AB) OR (take over request* KY))

28 ((driv* monitor* system*TI) OR (driv* monitor* system*AB) OR (driv* monitor* system*KY))

29 ((high* automat* driv* TI) OR (high* automat* driv* AB) OR (high* automat* driv* KY))

30 ((automat* driv* system* TI) OR (automat* driv* system* AB) OR (automat* driv* system* KY))

31 14 or 15 or 16 or 17 or 18 or 19 or 20 or 21 or 22 or 23 or 24 or 25 or 26 or 27 or 28 or 29 or 30 56902

32 ((fatigue TI) OR (fatigue AB) OR (fatigue KY))

33 ((task performance TI) OR (task performance AB) OR (task performance KY))

34 ((task engage* TI) OR (task engage* AB) OR (task engage* KY))

35 ((cognition TI) OR (cognition AB) OR (cognition KY))

36 ((stress TI) OR (stress AB) OR (stress KY))

37 (Eye Tracking CV)

38 (Eye Movements CV)

39 ((eye-tracking TI) OR (eye-tracking AB) OR (eye-tracking KY))

40 ((galvanic skin response TI) OR (galvanic skin response TI) OR (galvanic skin response KY))

41 (Skin Resistance CV)

42 ((skin conduct* TI) OR (skin conduct* AB) OR (skin conduct* KY))

43 ((electrodermal TI) OR (electrodermal AB) OR (electrodermal KY))

44 (Cardiovascular System CV)

45 ((heart rate TI) OR (heart rate AB) OR (heart rate KY))

46 ((attention TI) OR (attention AB) OR (attention KY))

47 (Human Reaction Time CV)

48 ((reaction time TI) OR (reaction time AB) OR (reaction time KY))

49 ((aware* TI) OR (aware* AB) OR (aware* KY))

50 (Risks CV)

51 ((bored* TI) OR (bored* AB) OR (bored* KY))

52 ((fixation TI) OR (fixation AB) or (fixation KY))

53 ((workload TI) OR (workload AB) OR (workload KY))

54 ((cognitive load TI) OR (cognitive load AB) or (cognitive load KY))

55 ((blood pressure TI) OR (blood pressure AB) or (blood pressure KY))

56 (Respiration CV)

57 ((respiration TI) OR (respiration AB) OR (respiration KY))

58 (Electromyography CV)

59 ((electromyograph* TI) OR (electromyograph* AB) OR (electromyograph* KY))

60 (Psychophysiology CV)

61 ((psychophysiolog* TI) OR (psychophysiolog* AB) OR (psychophysiolog* KY))

60 (Physiology CV)

61 ((physiolog* TI) or (physiology* AB) OR (physiology* KY))

62 ((drows* TI) OR (drows* AB) OR (drows* KY))

63 ((vigila* TI) OR (vigila* AB) OR (vigila* KY))

64 (Electrocardiography CV)

65 ((electrocardiograph* TI) OR (electrocardiograph* AB) OR (electrocardiograph* KY))

66 (Electroencephalography CV)

67 ((electroencephalograph* TI) OR (electroencephalograph* AB) OR (electroencephalograp* KY))

68 ((pupil* TI) OR (pupil* AB) OR (pupil* KY))

69 ((pupil* TI) OR (pupil* AB) OR (pupil* KY))

70 ((eyeblink TI) OR (eyeblink AB) OR (eyeblink KY))

71 ((PERCLOS* TI) OR (PERCLOS* AB) OR (PERCLOS* KY))

72 32 or 33 or 34 or 35 or 36 or 37 or 38 or 39 or 40 or 41 or 42 or 43 or 44 or 45 or 46 or 47 or 48 or 49 or 50 or 51 or 52 or 53 or 54 or 55 or 56 or 57 or 58 or 59 or 60 or 61 or 62 or 63 or 64 or 65 or 66 or 67 or 68 or 69 or 70 or 71

73 13 AND 31 AND 72

74 limit 73 to (english language and yr="2012 -Current")

Database: EMBASE (Elsevier)

1 'car'/de

2 automobil*:ab,ti,kw

3 ‘car driving’/de

4 ‘traffic accident’/de

5 ‘distracted driving’/de

6 driver impairment:ab,ti,kw

7 driver behavio*r:ab,ti,kw

8 driving performance:ab,ti,kw

9 human factors:ab,ti,kw

10 driver distraction:ab,ti,kw

11 driver take-over:ab,ti,kw

12 driver takeover:ab,ti,kw

13 1 or 2 or 3 or 4 or 5 or 6 or 7 or 8 or 9 or 10 or 11 or 12

14 ‘Automation’/de

15 ‘man machine interaction’/de

16 ‘Artificial Intelligence’/de

17 ‘Autonomous Vehicle’/de

18 autonomous vehicl*:ab,ti,kw

19 autonomous driving:ab,ti,kw

20 human automation interaction:ab,ti,kw

21 non-driving related task*:ab,ti,kw

22 vehicle automat*:ab,ti,kw

23 transition of control:ab,ti,kw

24 conditional* automat* driv*:ab,ti,kw

25 automat* driv*:ab,ti,kw

26 takeover request*:ab,ti,kw

27 take over request*:ab,ti,kw

28 driv* monitor* system*:ab,ti,kw

29 high* automat* driv*:ab,ti,kw

30 automat* driv* system*:ab,ti,kw

31 14 or 15 or 16 or 17 or 18 or 19 or 20 or 21 or 22 or 23 or 24 or 25 or 26 or 27 or 28 or 29 or 30

32 ‘Fatigue’/de

33 fatigue*:ab,ti,kw

34 ‘task performance’/de

35 task performance:ab,ti,kw

36 task engage*:ab,ti,kw

37 ‘Cognition’/de

38 ‘physiological stress’/de OR ‘psychological stress’/de

39 stress:ab,ti,kw

40 ‘Eye-Tracking’/de

41 eye-tracking:ab,ti,kw

42 ‘electrodermal response’/de

43 galvanic skin response:ab,ti,kw

44 skin conduct*:ab,ti,kw

45 electrodermal:ab,ti,kw

46 ‘heart rate’/de

47 heart rate:ab,ti,kw

48 ‘Attention’/de

49 attention:ab,ti,kw

50 ‘Psychomotor Performance’/de

51 ‘Reaction Time’/de

52 ‘Awareness’/de

53 aware*:ab,ti,kw

54 ‘high-risk behavior’/de

55 ‘Boredom’/de

56 bored*:ab,ti,kw

57 ‘eye fixation’/de

58 fixation:ab,ti,kw

59 ‘Workload’/de

60 cognitive load:ab,ti,kw

61 workload:ab,ti,kw

62 ‘Blood Pressure’/de

63 blood pressure:ab,ti,kw

64 ‘breathing’/de

65 respiration:ab,ti,kw

66 ‘Electromyography’/de

67 electromyograph*:ab,ti,kw

68 ‘Psychophysiology’/de

69 psychophysiolog*:ab,ti,kw

70 ‘Physiology’/de

71 physiolog*:ab,ti,kw

72 drows*:ab,ti,kw

73 vigila*:ab,ti,kw

74 electrocardiograph*:ab,ti,kw

75 electroencephalograph*:ab,ti,kw

76 pupil*:ab,ti,kw

77 blink*:ab,ti,kw

78 eyeblink:ab,ti,kw

79 PERCLOS*:ab,ti,kw

80 32 or 33 or 34 or 35 or 36 or 37 or 38 or 39 or 40 or 41 or 42 or 43 or 44 or 45 or 46 or 47 or 48 or 49 or 50 or 51 or 52 or 53 or 54 or 55 or 56 or 57 or 58 or 59 or 60 or 61 or 62 or 63 or 64 or 65 or 66 or 67 or 68 or 69 or 70 or 71 or 72 or 73 or 74 or 75 or 76 or 77 or 78 or 79

81 13 and 31 and 80

82 limit 81 to (english language and yr="2012 -Current")

Database: PsycINFO and PsycEXTRA (Ovid)

1 Automobiles/

2 automobil*.ab,ti,id.

3 Driving Behavior/ 21525

4 Motor traffic accidents/ 47044

5 Driver distraction/ 305

6 driver impairment.ab,ti,id. 59

7 driver behavio*r.ab,ti,id. 831

8 driving performance.ab,ti,id. 2021

9 human factors.ab,ti,id. 5416

10 driver distraction.ab,ti,id. 272

11 driver take-over.ab,ti,id. 5

12 driver takeover.ab,ti,id. 6

13 1 or 2 or 3 or 4 or 5 or 6 or 7 or 8 or 9 or 10 or 11 or 12 74475

14 Automation/ 19780

15 Human machine Systems/ 2899

16 Artificial Intelligence/ 33323

17 Autonomous Vehicles/ 80

18 autonomous vehicl*.ab,ti,id. 866

19 autonomous driving.ab,ti,id. 552

20 human automation interaction.ab,ti,id. 137

21 non-driving related task*.ab,ti,id. 49

22 vehicle automat*.ab,ti,id. 97

23 transition of control.ab,ti,id. 163

24 conditional* automat* driv*.ab,ti,id. 32

25 automat* driv*.ab,ti,id. 443

26 takeover request*.ab,ti,id. 18

27 take over request*.ab,ti,id. 31

28 driv* monitor* system*.ab,ti,id. 37

29 high* automat* driv*.ab,ti,id. 49

30 automat* driv* system*.ab,ti,id. 92

31 14 or 15 or 16 or 17 or 18 or 19 or 20 or 21 or 22 or 23 or 24 or 25 or 26 or 27 or 28 or 29 or 30 56902

32 Fatigue/ 33271

33 fatigue*.ab,ti,id. 116546

34 Task/ 32709

35 task performance.ab,ti,id. 13424

36 task engage*.ab,ti,id. 590

37 Cognition/ 119076

38 Psychological stress or Physiological stress/ 210608

39 stress.ab,ti,id. 905545

40 Eye Movements/ 651

41 eye-tracking.ab,ti,id. 7458

42 Galvanic Skin Response/ 8648

43 galvanic skin response.ab,ti,id. 977

44 skin conduct*.ab,ti,id. 5025

45 electrodermal.ab,ti,id. 2781

46 Heart Rate/ 173570

47 Attention/ 84251

48 attention*.ab,ti,id. 502284

49 Motor Performance/ 68163

50 Reaction Time/ 103003

51 Awareness/ 21588

52 aware*.ab,ti,id. 278350

53 Risk Taking/ 29442

54 Boredom/ 746

55 bored*.ab,ti,id. 2345

56 Eye Fixation/ 14309

57 fixation.ab,ti,id. 165311

58 Multitasking/ 183

59 Human channel capacity/ 23486

60 cognitive load.ab,ti,id.

61 workload.ab,ti,id. 30610

62 Blood Pressure/ 288140

63 blood pressure.ab,ti,id. 332657

64 Respiration/ 80797

65 respiration.ab,ti,id. 84995

66 Electromyography/ 83558

67 electromyograph*.ab,ti,id. 48899

68 Psychophysiology/ 9031

69 psychophysiolog*.ab,ti,id. 11563

70 Physiology/ 24197

71 physiolog*.ab,ti,id. 875400

72 drows*.ab,ti,id. 7540

73 vigila*.ab,ti,id. 24675

74 electrocardiograph*.ab,ti,id. 61675

75 electroencephalograph*.ab,ti,id. 49061

76 pupil*.ab,ti,id. 16127

77 blink*.ab,ti,id. 5335

78 eyeblink.ab,ti,id. 1775

79 PERCLOS*.ab,ti,id. 45

80 32 or 33 or 34 or 35 or 36 or 37 or 38 or 39 or 40 or 41 or 42 or 43 or 44 or 45 or 46 or 47 or 48 or 49 or 50 or 51 or 52 or 53 or 54 or 55 or 56 or 57 or 58 or 59 or 60 or 61 or 62 or 63 or 64 or 65 or 66 or 67 or 68 or 69 or 70 or 71 or 72 or 73 or 74 or 75 or 76 or 77 or 78 or 79 or 80

81 13 and 31 and 81

82 limit 80 to (english language and yr="2012 -Current")

Database: Web of Science (Core Collection)

1 Automobiles/ 7577

2 automobil$.ti,ab,kf. 8037

3 Automobile Driving/ 21525

4 Accidents, Traffic/ 47044

5 Distracted Driving/ 305

6 driver impairment.ab,ti,kf. 59

7 driver behavio*r.ab,ti,kf. 831

8 driving performance.ab,ti,kf. 2021

9 human factors.ab,ti,kf. 5416

10 driver distraction.ab,ti,kf. 272

11 driver take-over.ab,ti,kf. 5

12 driver takeover.ab,ti,kf. 6

13 1 or 2 or 3 or 4 or 5 or 6 or 7 or 8 or 9 or 10 or 11 or 12 74475

14 Automation/ 19780

15 Man-Machine Systems/ 2899

16 Artificial Intelligence/ 33323

17 Autonomous Vehicles/ 80

18 autonomous vehicl$.ab,ti,kf. 866

19 autonomous driving.ab,ti,kf. 552

20 human automation interaction.ab,ti,kf. 137

21 non-driving related task$.ab,ti,kf. 49

22 vehicle automat$.ab,ti,kf. 97

23 transition of control.ab,ti,kf. 163

24 conditional$ automat$ driv$.ab,ti,kf. 32

25 automat$ driv$.ab,ti,kf. 443

26 takeover request$.ab,ti,kf. 18

27 take over request$.ab,ti,kf. 31

28 driv$ monitor$ system$.ab,ti,kf. 37

29 high$ automat$ driv$.ab,ti,kf. 49

30 automat$ driv$ system$.ab,ti,kf. 92

31 14 or 15 or 16 or 17 or 18 or 19 or 20 or 21 or 22 or 23 or 24 or 25 or 26 or 27 or 28 or 29 or 30 56902

32 Fatigue/ 33271

33 fatigue$.ab,ti,kf. 116546

34 "Task Performance and Analysis"/ 32709

35 task performance.ab,ti,kf. 13424

36 task engage$.ab,ti,kf. 590

37 Cognition/ 119076

38 Stress, Physiological/ or Stress, Psychological/ 210608

39 stress.ab,ti,kf. 905545

40 Eye-Tracking Technology/ 651

41 eye-tracking.ab,ti,kf. 7458

42 Galvanic Skin Response/ 8648

43 galvanic skin response.ab,ti,kf. 977

44 skin conduct$.ab,ti,kf. 5025

45 electrodermal.ab,ti,kf. 2781

46 Heart Rate/ 173570

47 heart rate.ab,ti,kf.

48 Attention/ 84251

49 attention.ab,ti,kf. 502284

50 Psychomotor Performance/ 68163

51 Reaction Time/ 103003

52 Awareness/ 21588

53 aware$.ab,ti,kf. 278350

54 Risk-Taking/ 29442

55 Boredom/ 746

56 bored$.ab,ti,kf. 2345

57 Fixation, Ocular/ 14309

58 fixation.ab,ti,kf. 165311

59 Multitasking Behavior/ 183

60 Workload/ 23486

61 cognitive load.ab,ti,kf.

62 workload.ab,ti,kf. 30610

63 Blood Pressure/ 288140

64 blood pressure.ab,ti,kf. 332657

65 Respiration/ 80797

66 respiration.ab,ti,kf. 84995

67 Electromyography/ 83558

68 electromyograph$.ab,ti,kf. 48899

69 Psychophysiology/ 9031

70 psychophysiolog$.ab,ti,kf. 11563

71 Physiology/ 24197

72 physiolog$.ab,ti,kf. 875400

73 drows$.ab,ti,kf. 7540

74 vigila$.ab,ti,kf. 24675

75 electrocardiograph$.ab,ti,kf. 61675

76 electroencephalograph$.ab,ti,kf. 49061

77 pupil$.ab,ti,kf. 16127

78 blink$.ab,ti,kf. 5335

79 eyeblink.ab,ti,kf. 1775

80 PERCLOS$.ab,ti,kf. 45

81 32 or 33 or 34 or 35 or 36 or 37 or 38 or 39 or 40 or 41 or 42 or 43 or 44 or 45 or 46 or 47 or 48 or 49 or 50 or 51 or 52 or 53 or 54 or 55 or 56 or 57 or 58 or 59 or 60 or 61 or 62 or 63 or 64 or 65 or 66 or 67 or 68 or 69 or 70 or 71 or 72 or 73 or 74 or 75 or 76 or 77 or 78 or 79 or 80

82 13 and 31 and 81

83 limit 80 to (english language and yr="2012 -Current")

**Appendix B**

Listing of potential physiological measures that will potentially be found in included studies.

| Domain | Measure | Reference |
| --- | --- | --- |
| Electro-cardiac activity | Heart rate | Lohani M, Payne BR, Strayer DL. A review of psychophysiological measures to assess cognitive states in real-world driving. Frontiers in human neuroscience. 2019 Mar 19;13:57.  Charles RL, Nixon J. Measuring mental workload using physiological measures: A systematic review. Applied ergonomics. 2019 Jan 1;74:221-32. |
|  | R-R interval |  |
|  | SDNN (standard deviation of R-R intervals) |  |
|  | RMSSD (root mean squared of successive differences between adjacent R-R intervals) |  |
|  | pNN20 (% of successive R-R intervals >20ms) |  |
|  | pNN50 (% of successive R-R intervals >50ms) |  |
|  | LF (frequency activity in the 0.04 - 0.15Hz range) |  |
|  | HF (frequency activity in the 0.15 - 0.40Hz range) |  |
|  | LF/HF (ratio of LF to HF activity) |  |
| Respiration | Respiration rate | Lohani M, Payne BR, Strayer DL. A review of psychophysiological measures to assess cognitive states in real-world driving. Frontiers in human neuroscience. 2019 Mar 19;13:57. |
| Electrodermal activity | Phasic EDA (Skin conductance response) | Charles RL, Nixon J. Measuring mental workload using physiological measures: A systematic review. Applied ergonomics. 2019 Jan 1;74:221-32. |
|  | Tonic EDA (Skin conductance level) |  |
| Eye Movement | Pupil diameter | Kapitaniak B, Walczak M, Kosobudzki M, Jóźwiak Z, Bortkiewicz A. Application of eye-tracking in drivers testing: A review of research. International journal of occupational medicine and environmental health. 2015;28(6). |
|  | Number of gazes/gaze duration |  |
|  | Number of eyeblinks/eyeblink duration/eyeblink rate |  |
|  | Horizontal/vertical gaze dispersion |  |
|  | Total/average fixation duration/number of fixations |  |
|  | Eyes-on-road time |  |
|  | PERCLOS (percentage of eyelid closure over the pupil) |  |

| **Appendix C**  Data extraction form. | | | |
| --- | --- | --- | --- |
| Question category | Question | Response | Additional notes |
| Study ID |  |  |  |
| Bibliographic information | 1. study title  2. authors  3. year of publication  4. country of origin  5. Journal/conference |  |  |
| Study characteristics | 6. Study design (between subjects, within subjects…)  7. Theoretical background/conceptual models mentioned |  |  |
| Participant characteristics | 8. age  9. gender  10. experience with ADS |  |  |
| Experiment characteristics | 11. Type of vehicle or simulator used.  12. Type of NDRT(s) used  13. Duration of NDRT(s) used  14. Procedure of NDRT(s) |  |  |
| Outcome measures | 15. Type of physiological outcome measures  16. Outcome measurement methods |  |  |
| Findings | 17. Results with respect to physiological outcome measures |  |  |
| Limitations | 18. Limitations acknowledged by the authors |  |  |
